# Cervical Laminoforaminotomy for Treatment of Cervical Radiculopathy: A Case Report

**DOI:** 10.7759/cureus.65499

**Published:** 2024-07-27

**Authors:** Muhammad Syafik Mohd Yunus, Suffian Sabri, Sabarul A Mokhtar

**Affiliations:** 1 Department of Orthopaedics and Traumatology, Faculty of Medicine, Hospital Canselor Tuanku Muhriz, Universiti Kebangsaan Malaysia, Kuala Lumpur, MYS

**Keywords:** anterior cervical discectomy and fusion, open posterior cervical foraminotomy, minimally invasive posterior cervical foraminotomy, cervical lamino-foraminotomy, cervical radiculopathy

## Abstract

Cervical laminoforaminotomy (CLF) provides a safe and effective decompression procedure of nerve roots while maintaining cervical mobility and preserving stability. However, this unique technique requires appropriate patient selection and the technical ability of the surgeon to produce an excellent outcome. Furthermore, anterior cervical discectomy and fusion (ACDF) has been accepted as the “gold standard” procedure in managing cervical radiculopathy, despite posing the risk of anterior structure injuries and fusion. Here we report a case report of the surgical management of unilateral cervical radiculopathy using the CLF technique.

## Introduction

Cervical radiculopathy is a common condition that is typically caused by nerve root compression, either as a result of a herniated disc or spondylosis. Conservative treatments, such as medication and physical therapy, may temporarily relieve patients with cervical radiculopathy. However, in cases where patients have significant or worsening upper extremity weakness or do not respond to non-operative treatments, surgical interventions may be considered.

Cervical laminoforaminotomy (CLF) was first introduced in the 1940s and offers advantages in specific cases of cervical radiculopathy [1]. This procedure involves direct decompression of the nerve root while preserving cervical mobility and stability. The procedure is typically performed in patients with unilateral upper limb radiculopathy resulting from posterolateral disc herniation or disc osteophyte complex. Additionally, it can be used in situations where there has been incomplete anterior decompression after a previous anterior discectomy and fusion or disc arthroplasty procedure. CLF offers several advantages over anterior cervical discectomy and fusion [2]. This case report highlights the indications and benefits of CLF in treating cervical radiculopathy.

## Case presentation

A 38-year-old-lady with no known medical illness presented with an eight-week history of persistent neck pain and stiffness. Her symptoms worsened three weeks before presentation as she started to develop radicular pain, numbness over the left thumb, and reduced hand dexterity. These symptoms did not improve following conservative treatment, including medications and physiotherapy. She denied bladder or bowel incontinence, instability gait, and a recent history of trauma, smoking, or alcohol use.

Physical examination revealed tenderness over the mid-cervical region with left paraspinal muscle spasm and neck stiffness. There were no signs of myelopathy, but the Lhermitte and Spurling signs were positive. She had no motor deficit, with normal tone and reflexes. However, she had reduced sensation over the left thumb region and muscle atrophy over the left thenar region.

**Figure 1:**
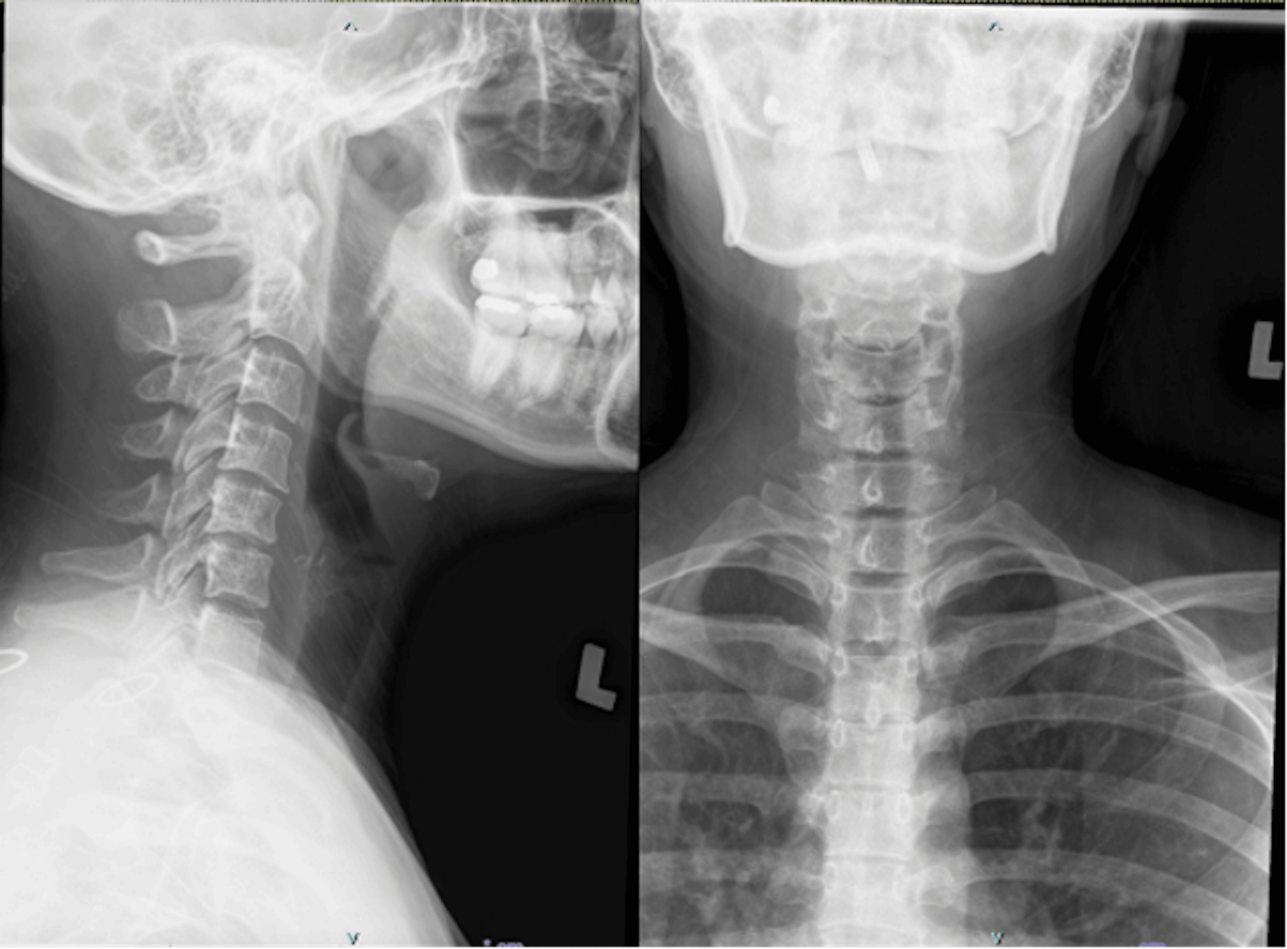
Plain radiographs of the cervical spine in lateral and anterior-posterior views showed reduced joint space at the C4/C5 and C5/C6 levels.

**Figure 2:**
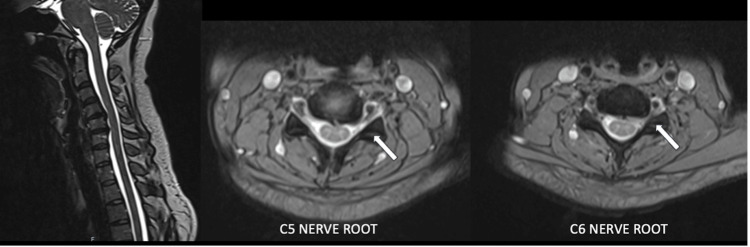
Preoperative MRI of the cervical spine in sagittal (left) and axial views of the C4/C5 (center) and C5/C6 levels (right), demonstrating foraminal stenosis with disc herniation at the left C5 and C6 nerve roots (arrows). MRI: magnetic resonance imaging.

Radiological findings of the plain cervical spine showed reduced joint space at the C4/C5 and C5/C6 levels (Figure 1). Magnetic resonance imaging (MRI) of the cervical spine showed circumferential disc osteophyte complex at the left C4/C5 and C5/C6, with posterior disc extrusion causing narrowing of the left neural foramina, suggestive of the left C5 and C6 exiting nerve root impingement (Figure 2).

**Figure 3:**
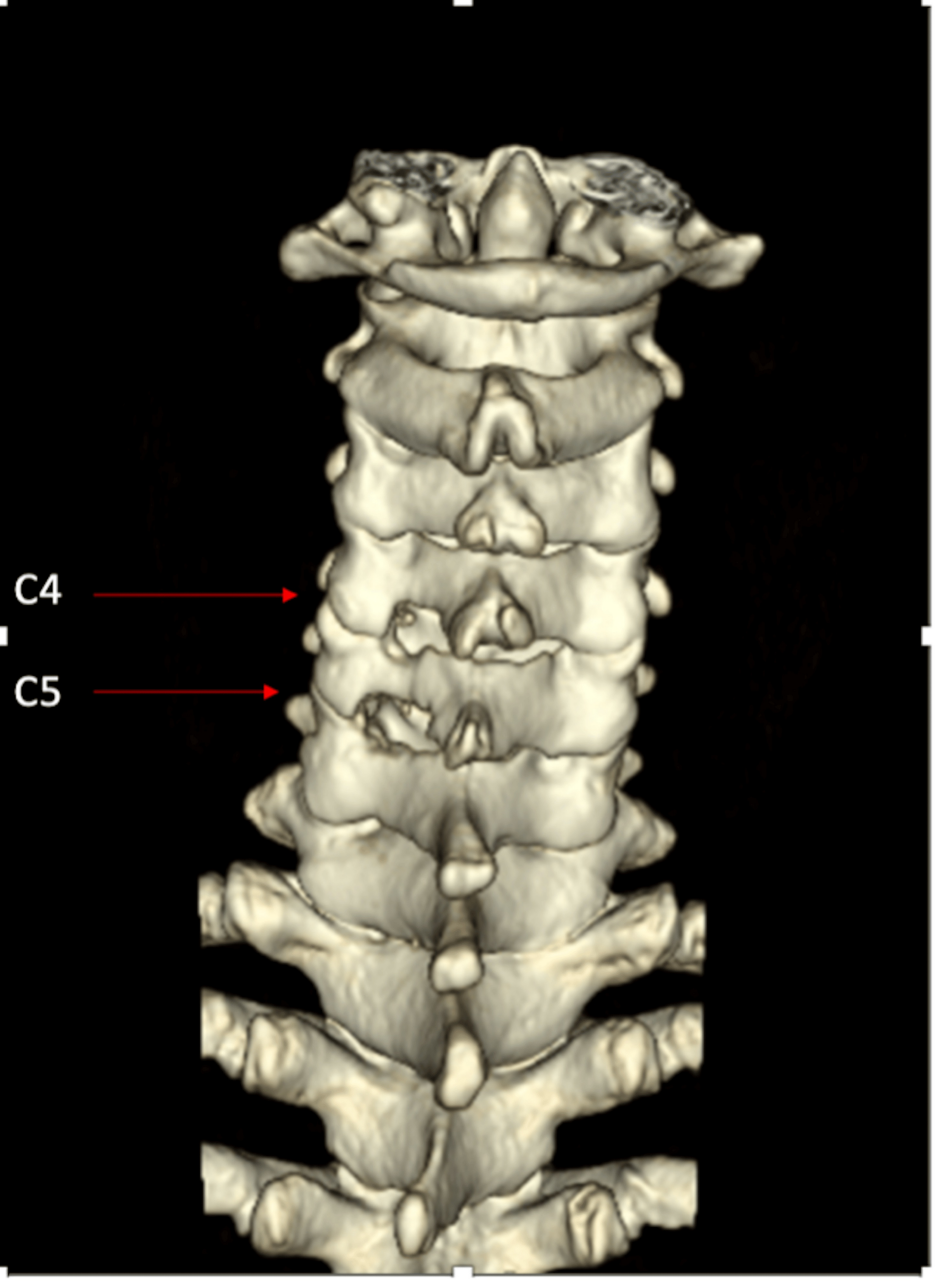
Postoperative 3D CT reconstruction image of the cervical spine, demonstrating the left C4/5 and C5/6 neural foramina are capacious after the left C4/5 and C5/6 laminoforaminotomy procedure (arrows).

**Figure 4:**
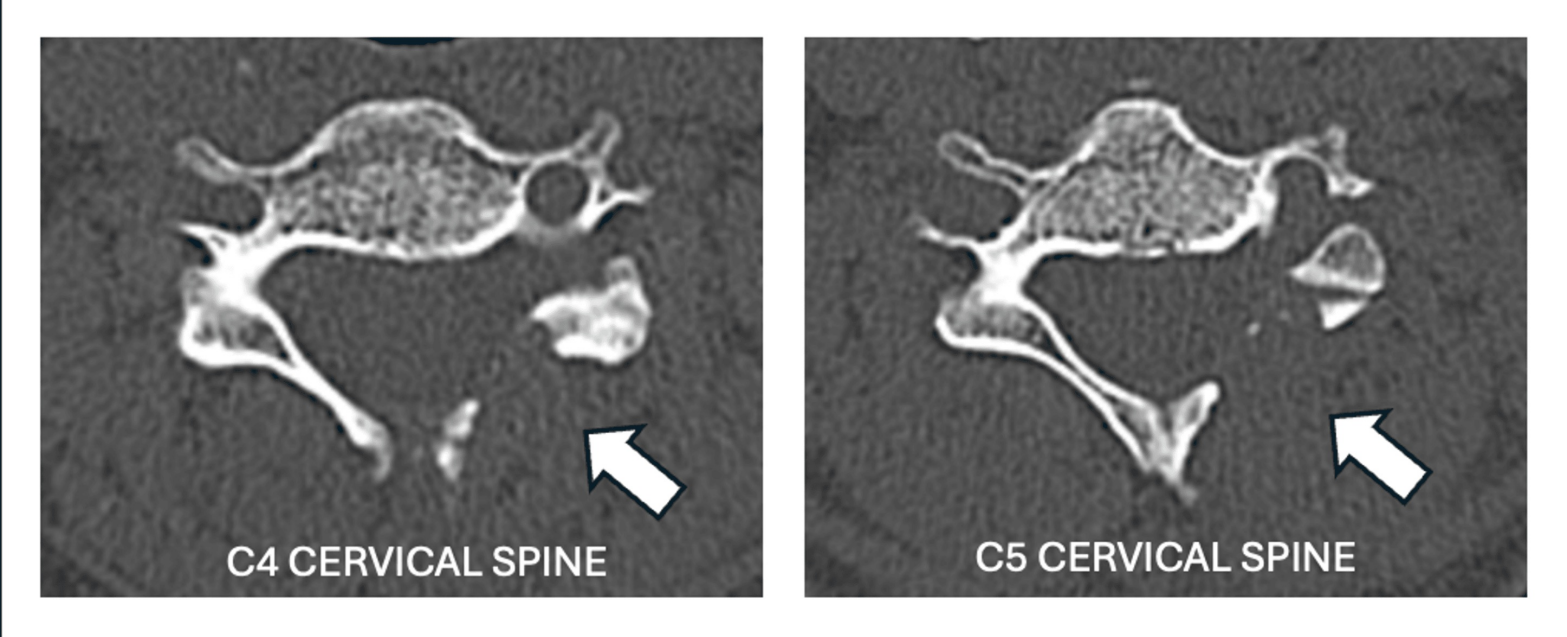
Postoperative CT scan in axial view at the C4 (left) and C5 cervical spine levels (right), demonstrating the left C4/5 and C5/6 neural foramina are capacious after the left C4/5 and C5/6 laminoforaminotomy procedure (arrows).

She underwent decompression of the left C5 and C6 exiting nerve roots by open CLF (Figures 3, 4). Intraoperative findings were consistent with the MRI findings, and we noticed pulsation and hyperemia of the nerve roots after decompression. The patient was stabilized in the ward post-operation without acute complications. The patient was discharged well the next day. Subsequent follow-up at three months postoperative noted that patient's symptoms had improved as evidenced by the absence of radicular pain, numbness, and hand dexterities, allowing her to return to her daily routine activities.

## Discussion

Anterior cervical discectomy and fusion (ACDF) and open CLF provide safe and effective surgical approaches for addressing cervical radiculopathy. Nevertheless, the results of these two surgical methods continue to be debated, and a definitive consensus on their superiority has yet to be reached.

Over the past two decades, ACDF has established itself as an efficient treatment for cervical radiculopathy. Despite numerous drawbacks in ACDF, it is still regarded as the preferred surgical method by many surgeons and is considered a gold standard. Conversely, CLF is not widely favored among surgeons and seems to have declined in prominence. Moreover, the rise in popularity of the minimally invasive surgery CLF (MIS-CLF) technique has led to a decline in the prominence of open cervical foraminotomy [3].

CLF presents a definitive surgical approach for addressing cervical radiculopathy. It provides direct nerve root decompression with easier access to the affected area. This allows for more efficient and targeted decompression, improving outcomes and faster recovery. Church et al. reported that a significant majority of patients experienced long-lasting improvements in pain (90.4%), weakness (88.6%), function (89.4%), and a high rate of returning to work (93%) after the procedure [4]. Although some surgeons argue that CLF can be technically demanding and suitable only for specific patients, its clinical outcome results are comparable to ACDF. A systematic review conducted by Liu et al. indicated that CLF demonstrates a success rate in pain relief ranging from 75% to 100%, which is comparable to the ACDF group (93.6-96%) [2]. Furthermore, cervical foraminotomy had an advantage over anterior cervical discectomy and fusion by preserving cervical mobility. Liu et al. reported that the postoperative range of motion (ROM) of the operated segment of the cervical spine was preserved in the CLF group but did not increase the ROM of the adjacent segment. It showed that the CLF has the ability to effectively decompress nerve roots while maintaining cervical mobility and preserving stability [2].

The debate concerning the complication outcomes of both approaches remains unresolved. These complications include issues related to implants and the anterior approach, such as dysphagia, hematoma, hoarseness, recurrent laryngeal nerve palsy, pseudoarthrosis, hardware failure, adjacent segment disease, and wound infection within the ACDF group. In the CLF group, complications include dermatome-related hyperesthesia, wound infection, and cerebrospinal fistula. However, Liu et al. found that the mean complication rate is lower in CLF (4%) compared to ACDF (7%), although there is no statistically significant difference. Furthermore, they also found that the reoperation rates between these two groups did not exhibit a statistically significant difference. The mean reoperation rates within two years were 4% in the ACDF group and 6% in the CLF group. They concluded with a strong level of recommendation that no difference existed in clinical outcome, complication rate, and reoperation rate between the ACDF and CLF groups. However, CLF offers the advantage of cost-effectiveness and reduces the incidence of adjacent segment disease [2].

Meanwhile, CLF has limitations as this procedure was exclusively applied to specific patients. Ideally, the patient should present with a soft lateral disc herniation that results in nerve root compression and concurrent radicular pain without severe cervical spondylosis. In this particular instance, the patient experienced cervical radiculopathy due to disc herniation and osteophyte disease. These conditions were initially diagnosed through MRI and later confirmed during surgery. In her situation, substantial neck and arm pain were alleviated after undergoing CLF. Besides, CLF is contraindicated in cases when flexion-extension X-rays reveal cervical instability. It is important to note that this particular approach is not recommended for patients presenting with central stenosis caused by diffuse spondylosis. This is due to the fact that the spinal cord is not sufficiently decompressed when employing this technique. Moreover, a patient with a diffuse anterior disc or osteophyte is better treated with ACDF rather than cervical foraminotomy, as the disease is readily accessible via an anterior route. Further contraindications for this treatment include bilateral radicular complaints, previous ipsilateral foraminotomy, and lateral mass hypoplasia, in which additional decompression may worsen cervical instability [1].

CLF can be performed following an open approach or MIS approach using a tubular retractor system. A meta-analysis found that there was no significant difference in clinical outcomes when patients with symptomatic cervical radiculopathy caused by foraminal stenosis underwent open or MIS foraminotomy [5]. Recently, many surgeons favored the MIS approach, as they believe that MIS-CLF theoretically offers advantages over open CLF in terms of reduced estimated blood loss (EBL), pain, and hospital stay duration. Nevertheless, adopting the MIS-CLF approach with the use of an operating microscope is already a technically demanding procedure and carries the risk of undesirable complications, such as incomplete decompression and the potential for neurological injury due to limited exposure. The risk of infections, dura tears, and cerebrospinal fluid fistula can also occur, although these issues have been underreported in the literature [3].

## Conclusions

CLF can provide decompression for foraminal nerve root stenosis in appropriately selected patient. This procedure has a lower risk of complications, including avoiding potential injury to anterior cervical structures and preserving cervical mobility and stability with an excellent clinical outcome.
